# Development of Commercial Thermo-sensitive Genic Male Sterile Rice Accelerates Hybrid Rice Breeding Using the CRISPR/Cas9-mediated *TMS5* Editing System

**DOI:** 10.1038/srep37395

**Published:** 2016-11-22

**Authors:** Hai Zhou, Ming He, Jing Li, Liang Chen, Zhifeng Huang, Shaoyan Zheng, Liya Zhu, Erdong Ni, Dagang Jiang, Bingran Zhao, Chuxiong Zhuang

**Affiliations:** 1 State Key Laboratory for Conservation and Utilization of Subtropical Agro-bioresources, Guangzhou 510642, China; 2 Key Laboratory of Plant Functional Genomics and Biotechnology of Guangdong Provincial Higher Education Institutions, Guangzhou 510642, China; 3 College of Life Sciences, South China Agricultural University, Guangzhou 510642, China; 4 State Key Laboratory of Hybrid Rice, China National Hybrid Rice R&D Center, Changsha 410125, China

## Abstract

Hybrid rice breeding offers an important strategy to improve rice production, in which the cultivation of a male sterile line is the key to the success of cross-breeding. CRISPR/Cas9 systems have been widely used in target-site genome editing, whereas their application for crop genetic improvement has been rarely reported. Here, using the CRISPR/Cas9 system, we induced specific mutations in *TMS5*, which is the most widely applied thermo-sensitive genic male sterility (TGMS) gene in China, and developed new “transgene clean” TGMS lines. We designed 10 target sites in the coding region of *TMS5* for targeted mutagenesis using the CRISPR/Cas9 system and assessed the potential rates of on- and off-target effects. Finally, we established the most efficient construct, the TMS5ab construct, for breeding potentially applicable “transgene clean” TGMS lines. We also discussed factors that affect the editing efficiency according to the characteristics of different target sequences. Notably, using the TMS5ab construct, we developed 11 new “transgene clean” TGMS lines with potential applications in hybrid breeding within only one year in both rice subspecies. The application of our system not only significantly accelerates the breeding of sterile lines but also facilitates the exploitation of heterosis.

Rice (*Oryza sativa* L.) is one of the most important staple foods worldwide, providing almost one-quarter of the global dietary energy supply for humans^1^,^2^. The demand for food continues to rise as the population rapidly grows, necessitating the production of 40% more rice in 2030, and arable land is limited together with environmental degradation^3^. Hybrid rice, which has been developed in more than 40 countries worldwide and plays a key role in the global food supply^4^, has a 10–20% yield advantage over conventional rice and occupies approximately 60% of the total rice area in China^5^,^6^. The hybrid rice developed using the three- and two-line hybrid breeding systems dominates hybrid rice production in China^7^,^8^. The three-line system utilizes a cytoplasmic male-sterile (CMS) line, a restorer line and a maintainer line to produce hybrid seeds and to maintain the CMS line^8^,^9^. The restorer line carries special CMS-restorer genes to restore the fertility of the special CMS line. The germplasm resources related to cytoplasmic sterility are limited: only 1% of rice germplasms can serve as maintainer lines in China, and only 5% of rice germplasms carry CMS-restorer genes in Southeast Asia^10^. The limited genetic resources of the restorer lines and the low genetic biodiversity of CMS and restorer lines in the three-line system have prevented further developments. The two-line breeding system uses either photoperiod-sensitive genic male-sterile (PGMS) or thermo-sensitive genic male-sterile (TGMS) lines as sterility lines under the restrictive condition or maintainer lines under the permissive condition. Almost all normal rice cultivars can restore the male fertility of the PGMS and TGMS lines, thus providing broader genetic resources to better exploit heterosis in rice^11^,^12^,^13^,^14^,^15^. Therefore, compared with the three-line system, the advantages of the two-line system include labour and time savings, better grain quality and higher yields, greater effectiveness and economical use of simpler procedures for breeding and hybrid seed production. Although the two-line hybrid breeding system was developed relatively late, it provides essential advantages over the three-line system and occupies approximately one-third of the total hybrid rice planting area in China^16^.

In recent years, great progress has been achieved in the understanding of P/TGMS, and several genes that control P/TGMS traits have been cloned in rice. Nongken58S, the first PGMS rice identified in 1973, is characterized by complete male sterility under long-day conditions and fertility recovery under short-day conditions. Its PGMS trait is determined by *pms1, pms2* and *pms3* (refs 12 and 17). *pms3* encodes a long non-coding RNA (lncRNA) called *long day-specific male fertility-associated RNA* (*LDMAR*)^18^. Peiai64S is a TGMS *indica* rice line that was developed by transferring P/TGMS genes from Nongken58S. Its TGMS trait is conferred by *p/tms12-1*. We found *P/TMS12-1* encodes a unique noncoding RNA that produces a 21-nucleotide small RNA^13^. *Carbon Starved Anther* (*csa*) is a reverse photoperiod-sensitive genic male sterile (rPGMS) rice that is sterile under short day and fertile under long day conditions. *CSA* encodes a R2R3 MYB transcription regulator that mediates sugar partitioning^14^. In addition, temperature-sensitive splicing of *UDP-glucose pyrophosphorylase1* (*Ugp-1*) caused by *Ugp-1* overexpression leads to TGMS in rice^19^.

Annong S-1 (AnS-1) was the first *indica* TGMS rice identified in 1987. In a previous work^15^, we demonstrated that the TGMS gene *tms5* encodes the endonuclease RNase Z^S1^ in AnS-1. *RNase Z*^*S1*^ controls the TGMS trait by degrading the temperature-sensitive *ubiquitin fusion ribosomal protein L40* (*Ub*_*L40*_) mRNA. The *tms5-*determined TGMS lines play a major role in the two-line hybrid breeding system in China^20^. Mutations in *TMS5* were found in 24 of 25 commercial TGMS lines that were randomly detected^15^.

While the development of a new commercial male sterile line using traditional breeding systems usually takes several years, sometimes more than a decade, the breeding time can be dramatically reduced using modern techniques of genetic engineering^21^,^22^,^23^,^24^. Sequence-specific nucleases (SSNs) can induce targeted DNA double-stranded breaks (DSBs) at specific genomic loci and promote endogenous pathways of DNA damage repair, finally leading to sequence-specific genome editing^25^,^26^. As a type of SSN, the CRISPR/Cas9 editing system has been harnessed to knock out the targeted gene in many species, including plants^25^,^26^,^27^,^28^,^29^,^30^,^31^,^32^,^33^,^34^,^35^,^36^,^37^,^38^. Nevertheless, its application for crop genetic improvement remains rare^39^.

In this study, we established a simple and efficient rice TGMS cultivating system using CRISPR/Cas9 editing technology to knock out *TMS5*, which is of great value in new commercial TGMS line applications. Based on this system, we developed 11 commercial “transgene clean” TGMS rice lines within only one year. This work accelerates TGMS line breeding and lays the foundation for large-scale applications in two-line hybrid rice breeding.

## Results

### Development of TGMS lines using CRISPR/cas9 editing of *TMS5*

**Figure 1:**
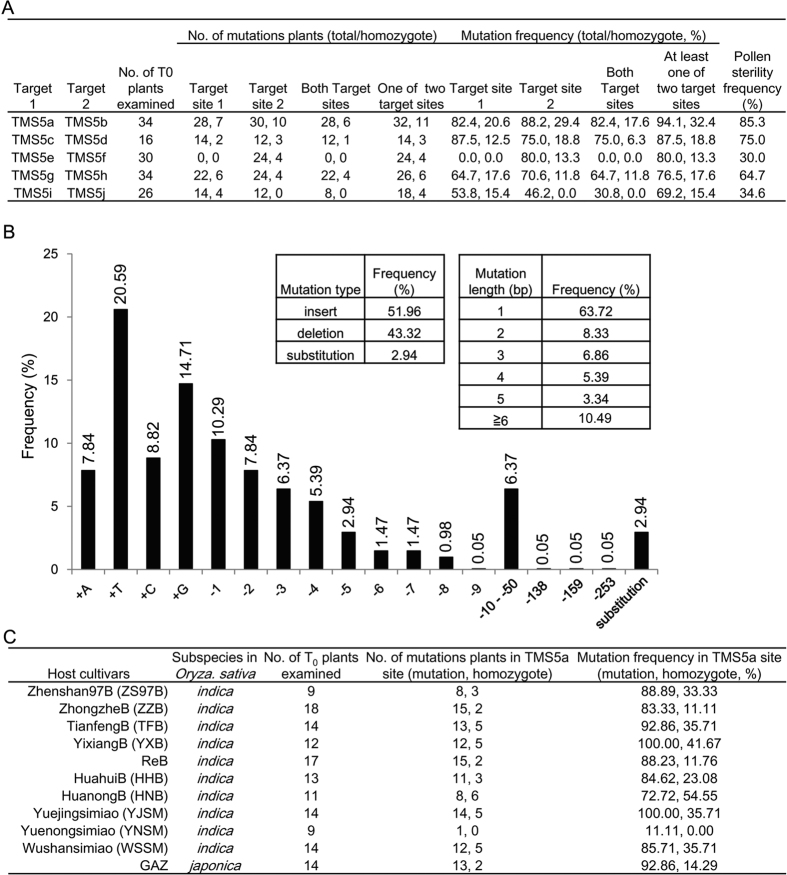
Mutation types and frequencies in T_0_ plants with 10 target sites. (**A**) Mutation and pollen sterility frequencies in T_0_ plants with 10 target sites. The target site of TMS5b displayed the highest mutation frequency. The TMS5ab construct gave rise to a higher percentage of plants with pollen sterility compared to the other constructs. The number preceding “,” is the total number or frequency of mutations, whereas the number following “,” is the number or ratio of homozygote plants. (**B**) CRISPR/Cas9-induced mutation types and frequencies. In all types of induced mutations, single-nucleotide insertions were most frequently detected. The largest deletion was 253 base pairs long. (**C**) TMS5ab construct-induced mutation frequencies in 11 cultivars of two different rice subspecies. Excluding YNSM, the mutation frequencies of TMS5a in the other ten cultivars were all higher than 72.72%.

The CRISPR/Cas9 editing system mediates targeted genome editing through the complex of Cas9 endonuclease and guide RNA, which provides a simple and efficient technique compared with other genome engineering technologies, such as zinc-finger nucleases (ZFNs) and transcription activator-like effector nucleases (TALENs)^39^,^40^. CRISPR/Cas9-induced editing events take place mainly in the T-DNA transformed callus cells before regeneration in rice^26^. Therefore, homozygous mutants with high mutation frequencies can be obtained in the T_0_ generation, and mutants without exogenous T-DNA can be isolated after segregation of the T_1_ generation. These mutants are also named “transgene clean” plants^41^. We used the CRISPR/cas9 editing system, which is able to target multiple sites via a single construct^26^, to create *TMS5-*targeted mutations to develop commercial TGMS lines. To obtain target sequences with high editing and low off-target efficiencies, 10 target sites (Supplementary Figure 1) screened from the *TMS5* coding region were randomly divided into five groups, and the two target sequences in one group were cloned into the same vector to transform the ZH11 callus (see Methods). Transgenic plants were obtained based on five vectors. To evaluate the editing efficiencies of each target sequence, the target sites in the leaves of T_0_ plants were sequenced. Mutations were detected in 9 of 10 target sites. Among these nine mutation sites, the single mutation frequencies of the target sequence ranged from 46.2% to 88.2% and from 69.2% to 94.1% T_0_ plants contained mutations in at least one of the two target sites (Fig. 1A). Notably, the homozygotic mutation frequency for single target sequences reached 29.4% and the plants carrying homozygous mutations for at least one of two target sequences reached 32.4% (Fig. 1A). Furthermore, individuals with pollen sterility were obtained for all T_0_ plants under high-temperature (HT) treatment. The percentages of pollen-sterile individuals among the T_0_ plants ranged from 30.0% to 85.3% (Fig. 1A). TMS5b showed the highest editing, homozygous mutation, and pollen sterile frequencies (Fig. 1A). The above results indicated that the binary construct for targeting the TMS5a and TMS5b sites (named the TMS5ab construct) had the highest targeted editing efficiency.

**Figure 2:**
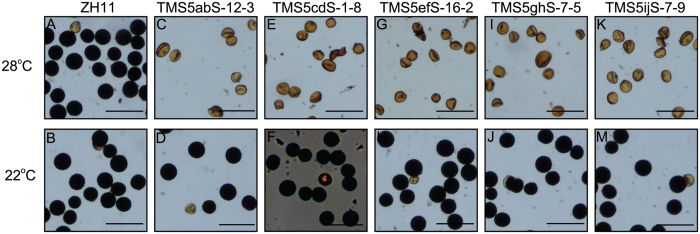
Pollen fertility of TGMS lines in the ZH11 background induced by CRISPR/Cas9 systems at high and low temperatures. (**A**,**B**) Pollen fertility of ZH11. Normal pollen (**A**) in ZH11 at the HT. Normal pollen (**B**) in ZH11 at the LT. (**C**–**M**) Pollen fertility of TGMS plants induced by the CRISPR/Cas9-mediated *TMS5* editing system. Abnormal pollen (**C**,**E**,G,**I** and **K**) in TMS5abS-12-3, TMS5cdS-1-8, TMS5efS-16-2, TMS5ghS-7-5 and TMS5ijS-7-9 at the HT. Normal pollen (**D**,**F**,**H**,**J** and **M**) in TMS5abS-12-3, TMS5cdS-1-8, TMS5efS-16-2, TMS5ghS-7-5 and TMS5ijS-7-9 at the LT. TMSabS-12-3, TMS5cdS-1-8, TMS5efS-16-2, TMS5ghS-7-5 and TMS5ijS-7-9 are TGMS plants induced by the TMS5ab, TMS5cd, TMS5ef, TMS5gh and TMS5ij constructs in the ZH11 background, respectively. The growth temperatures and plant names of the pollen are indicated on the left and top of the figure, respectively. HT, high temperature; LT, low temperature. Scale bars, 100 μm.

**Figure 3:**
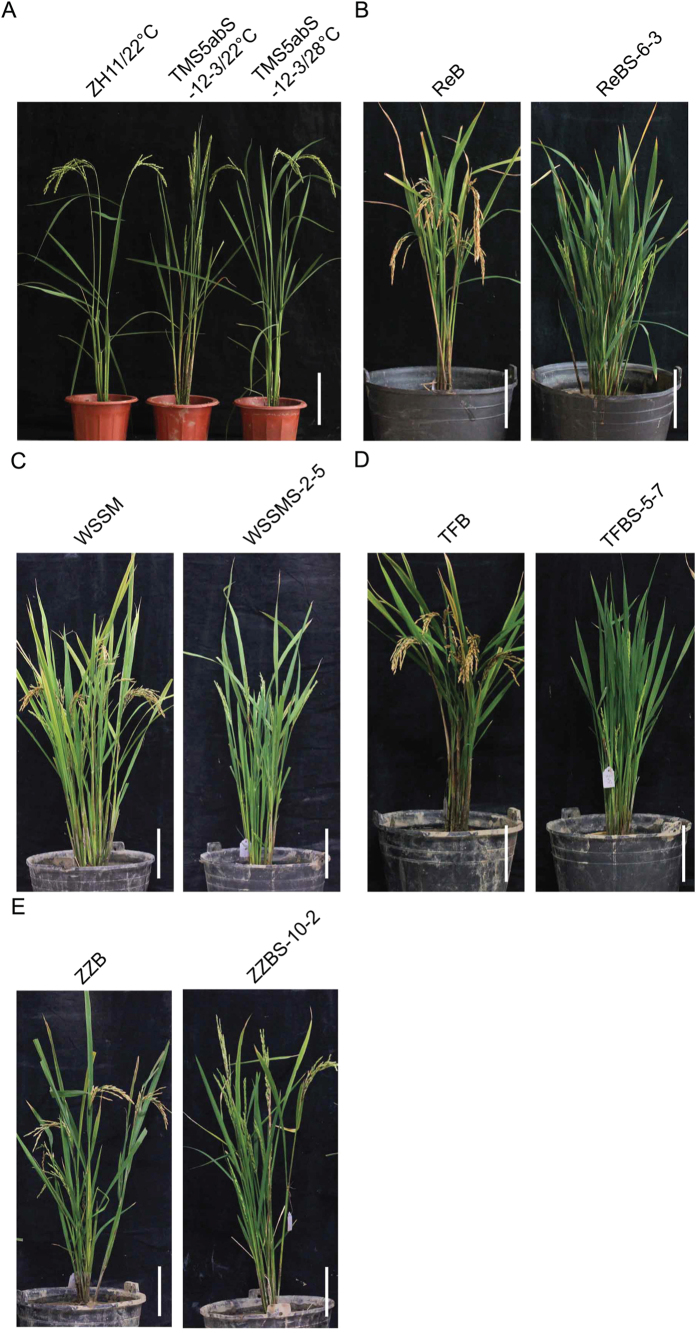
Plant morphologies of wild-type and TGMS plants at HT. (**A**) Plant morphologies of ZH11 at the HT and TMS5abS-12-3 plants at the HT and LT. (**B**) Plant morphologies of ReB and ReBS-6-3 plants at the HT. (**C**) Plant morphologies of WSSM and WSSMS-2-5 plants at the HT. (**D**) Plant morphologies of TFB and TFBS-5-7 plants at the HT. (**E**) Plant morphologies of ZZB and ZZBS-10-2 plants at the HT. While exhibiting pollen sterility, these “transgene clean” TGMS plants had no obvious phenotypic variation from their hosts. HT, high temperature; LT, low temperature. Scale bars, 20 cm.

To obtain TGMS plants, T_0_ sterile plants were treated at a low temperature (~22 °C, daily average temperature [DAT]). Plants with restored fertility were obtained from tranformants of each construct. Furthermore, we obtained “transgene clean” TGMS lines in T_1_ plants with each construct (Figs 2 and 3A and Supplementary Figure 2A). Taken together, we obtained “transgene clean” TGMS lines through the CRISPR/Cas9-mediated *TMS5* site-directed mutagenesis system.

Previous reports have shown that CRISPR/Cas9 technology has a relatively high potential for off-target activity, which affects its application. The specificity of the seed sequence, including the 10–12 nucleotides close to the protospacer adjacent motif (PAM), has a significant impact on the off-target activity^42^. The TMS5ab construct has the highest efficiency among the constructs used for CRISPR/Cas9-mediated *TMS5* editing; therefore, we analysed the off-target efficiency of TMS5ab. We predicted five and nine potential off-target sites for TMS5a and TMS5b, respectively. After sequencing those potential off-target sites, no genome editing was detected in any examined samples (Table 1). These results indicated that the TMS5ab construct had the highest editing efficiency and an undetectable off-target effect. The target sequences with high editing and low off-target efficiencies will improve the breeding efficiency and reduce the breeding cost associated with the development of commercially applied TGMS lines. Therefore, we exploited the TMS5ab construct for the transformation of other rice varieties for commercially applied TGMS lines.

### Editing characteristic of *TMS5* and factors influencing editing efficiency

Next, we analysed the mutation types and frequencies of the nine effective target sequences (Fig. 1B). Among all types of induced mutations, single-nucleotide insertions were most frequently detected, with a frequency up to 51.96%, consistent with previous reports^25^,^26^. The second highest frequency of mutation was nucleotide deletion, in which single-nucleotide deletions showed the highest mutation rate of 10.29%. Together with the increased number of deleted nucleotides, the mutation frequency of the target site became gradually reduced. The longest deletion fragment was 253 bp in length. Nucleotide substitutions were also observed, accounting for only 2.94% of all mutations.

**Figure 4:**
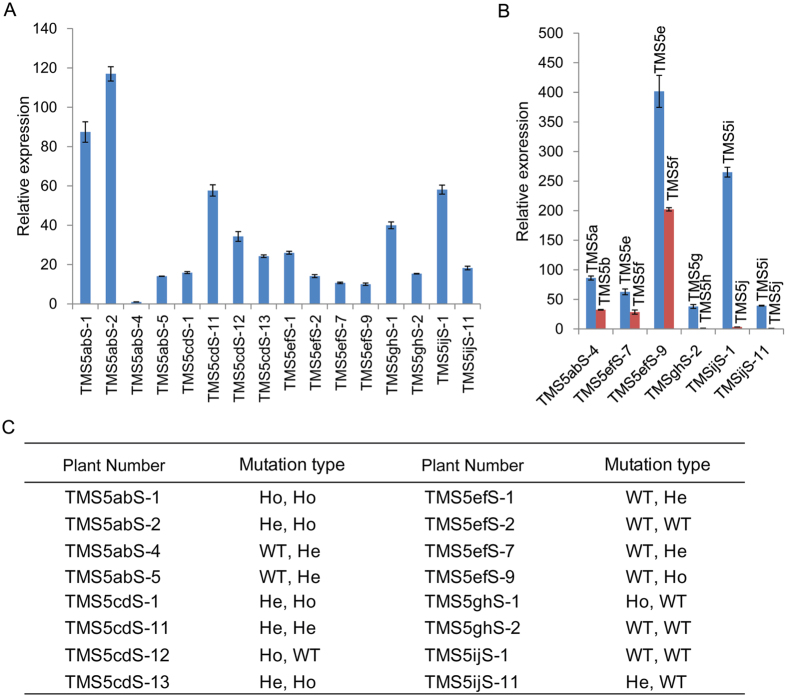
Effects of *Cas9* and target-sgRNA expression levels on target site editing. (**A**) *Cas9* expression levels in T_0_ plants (TMS5abS-1, TMS5abS-2, TMS5abS-4, TMS5abS-5, TMS5cdS-1, TMS5cdS-11, TMS5cdS-12, TMS5cdS-13, TMS5efS-1, TMS5efS-2, TMS5efS-7, TMS5efS-9, TMS5ghS-1, TMS5ghS-2, TMS5ijS-1 and TMS5ijS-11) assessed by real-time PCR analysis. (**B**) Target-sgRNA expression levels in T_0_ plants (TMS5abS-4, TMS5efS-7, TMS5efS-9, TMS5ghS-2, TMS5ijS-1 and TMS5ijS-11) assessed by real-time PCR analysis. The target sequence names are indicated above the column. (**C**) The mutation types in T_0_ plants (TMS5abS-1, TMS5abS-2, TMS5abS-4, TMS5abS-5, TMS5cdS-1, TMS5cdS-11, TMS5cdS-12, TMS5cdS-13, TMS5efS-1, TMS5efS-2, TMS5efS-7, TMS5efS-9, TMS5ghS-1, TMS5ghS-2, TMS5ijS-1 and TMS5ijS-11). He, heterozygote; Ho, homozygote; WT, wild type.

Next, we analysed the factors that affect editing efficiency. First, we analysed the GC content of the target sequences because targeted genome editing relies on the binding of the single-guide RNA (sgRNA) containing the target sequence to the target sites. By comparing the GC contents in the target sequences and the corresponding editing efficiency, we found that a high GC content is correlated with a high target sequence editing efficiency (Fig. 1A and Supplementary Figure 3). We then proceeded to test the sgRNA and *Cas9* expression levels from the leaves of T_0_ plants carrying different target sequences (Fig. 4). The *Cas9* expression levels were lowest in TMS5abS-4 plants, which displayed modified TMS5b target sites and no modified TMS5a sites. In contrast, the *Cas9* expression levels in TMS5efS-2, TMS5ghS-2 and TMS5ijS-1 plants were 14.1, 15.3 and 58.1 times larger than that in TMS5abS-4, respectively. However, no genome editing occurred in any of the target sites in these three lines. A similar phenomenon was also observed for the relationship between the target and the sgRNA and the editing efficiency. This finding suggested that once the sgRNA and *Cas9* expression levels reach a certain threshold value, there is no obvious correlation between their expression levels and the editing efficiencies. Furthermore, because PAM is essential for identification of the target site by the target sequence-sgRNA, we analysed the effects of different PAMs on the editing efficiencies of the target sites (Fig. 1A and Supplementary Table 1). The average editing efficiencies of the target site PAMs with AGG (TMS5c, d and f), TGG (TMS5b, g, h and j), and CGG (TMS5a and i) were 80.6%, 68.8%, and 70%, respectively. Although AGG displayed a higher average editing efficiency compared with TGG and CGG, TMS5b, which contained TGG as the PAM, displayed the highest editing efficiency among all nine target sequences. These findings indicated that the type of target sequence PAMs had no significant effect on the editing efficiency. We also found that the TMS5e target sequence was highly complementary to the sgRNA, forming a secondary structure that was likely to inhibit binding of the target sequence to the genome (Supplementary Figure 4). This was the plausible cause of the genome editing failure and is supported by previous studies^25^,^26^.

### Development of commercial TGMS lines in rice

The TMS5ab construct established in our study showed promising utilization potential. To develop the applied TGMS lines, we selected 11 fertile elite cultivars of two different rice subspecies (including seven maintainer lines of the three-line system, three *indica* and one *japonica* conventional variety) as the hosts for the targeted genome editing using the TMS5ab construct over the course of only one year (Fig. 1C). We obtained various numbers of T_0_ transgenic plants in all 11 cultivars. Excluding the identification of only one YNSMS (Yuenongsimiao (YNSM) transformed with TMS5ab) plant carrying the edited TMS5a, the editing efficiencies among the other cultivars ranged from 72.72% to 100%. While no homozygous TMS5a mutation was found in YNSMS, the homozygous mutation rates of the other cultivars ranged from 11.1% to 54.55%. These results indicated that we had produced *TMS5-*targeted mutation plants in all 11 cultivars and that the editing efficiency of the CRISPR/Cas9 system may vary depending on the genetic backgrounds of the different cultivars. After LT treatment of the T_0_ sterile individuals, plants with restored fertility were selected.

**Figure 5:**
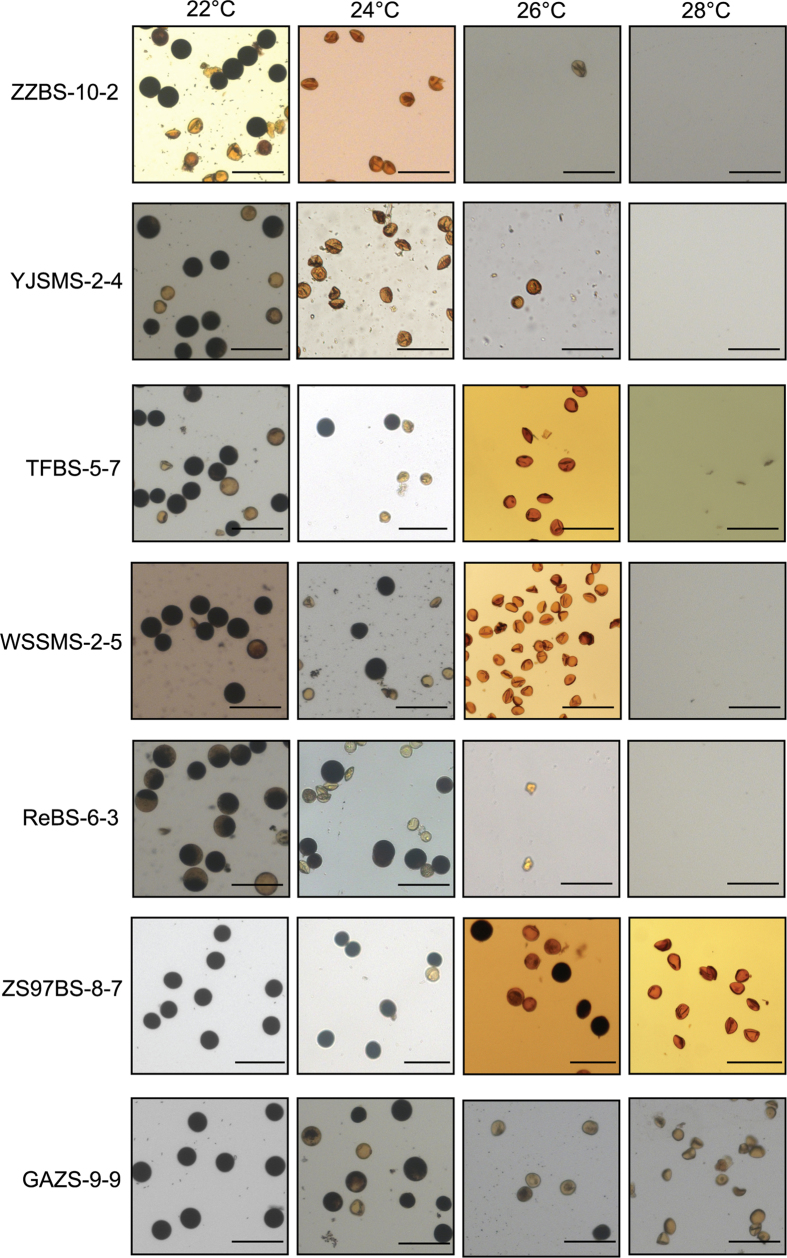
Pollen fertility of TGMS lines induced by the TMS5ab construct at different temperatures. ZZBS-10-2, ReBS-6-3, TFBS-5-7, WSSMS-2-5, YJSMS-2-4, ZS97BS-8-7 and GAZS-9-9 are the TGMS plants induced by the TMS5ab construct in the ZZB, ReB, TFB, WSSM, YJSM, ZS97B and GAZ backgrounds, respectively. These TGMS plants were grown at 22 °C, 24 °C, 26 °C, and 28 °C under photoperiod conditions of 13.5 h of light and 10.5 h of darkness. ZZBS-10-2 and YJSMS-2-4 plants were completely sterile at 24 °C (DAT); ReBS-6-3, TFBS-5-7 and WSSMS-2-5 plants were almost sterile at 24 °C (DAT); GAZS-9-9 plants were sterile at approximately 26 °C (DAT); and ZS97BS-8-7 plants were sterile at greater than 26 °C (DAT). The TGMS plant names and growth temperatures of the pollen are indicated at the left and top of the figure, respectively. Scale bars, 100 μm.

To obtain TGMS plants without exogenous T-DNA, plants with restored fertility from 11 cultivars were planted under high temperature (HT) conditions. Additionally, sterile plants without hygromycin phosphotransferase (*HPT*) and *Cas9* were obtained by PCR and Southern blot detection (Supplementary Figure 2B,C and Supplementary Table 2). These “transgene clean” sterile plants were grown under LT conditions, and T_2_ seeds were obtained. The “transgene clean” TGMS lines segregated in the T_1_ generation. While exhibiting similar pollen sterility, these “transgene clean” TGMS plants displayed no obvious phenotypic changes compared with their hosts (Fig. 3B–E and Supplementary Figure 5B). The phenotypes of these TGMS lines as a consequence of genome editing were stable in the T_2_ and T_3_ generations. To obtain TGMS lines that were applicable to two-line hybrid breeding, we next treated these TGMS lines with different temperature gradients and found that fertility was gradually reduced with increasing temperature (Fig. 5). TGMS lines of ZZBS and YJSMS showed relatively low critical sterility-inducing temperature (CSIT), i.e., below 24 °C (DAT), while in the ReBS, TFBS and WSSMS TGMS lines, the CSIT were all about 24 °C (DAT). The CSIT of the GAZS TGMS lines was approximately 26 °C (DAT), and the CSIT of ZS97BS exceeded 26 °C (DAT). ZZBS, ReBS, TFBS, WSSMS and YJSMS exhibited a pollenless sterile type, whereas ZS97BS and GAZS displayed completely abortive pollen at 28 °C (DAT). These results demonstrated that CSIT was regulated by the rice genetic background. The populations of “transgene clean” YJSMS were grown in the experimental plots of the China National Hybrid Rice R&D Center (Changsha, China) in the summer of 2015 and they displayed pollenless male sterility (Supplementary Figure 5). To confirm that *TMS5* editing would not lead to female organ abortion, GAZS and YJSMS were pollinated with normal pollen. The sterile plants of GAZS and YJSMS yielded seed settings via artificial pollination (Supplementary Figure 6), indicating normal female organ development and fertilization functions. These results indicated that these lines could be used as male sterile lines, which are almost indistinguishable from traditional male sterile lines. Taken together, the findings suggest that practical TGMS lines with good agronomic characteristics can be developed by targeted editing of *TMS5* using the CRISPR/Cas9 system.

To test the combinatorial capacity of the obtained TGMS lines, we crossed HNBS with Huahang1179 (HH1179), YJSMS with R94, YJSMS with R340, YJSMS with R297, YJSMS with R173, WSSMS with R364, WSSMS with R173, WSSMS with R340, and WSSMS with R297. Subsequently, we obtained the F_1_ generation of HNBS and HH1179 and found that the offspring were stronger and provided a greater yield than those of the control variety (Supplementary Figure 7). In addition, the hybrid demonstrated a 31.4% increase in the main panicle length, a 47.9% increase in grain number per main spike, a 19.8% increase in thousand seed weight, a 14.4% increase in single plant yield, and a 15.9% increase in plot yield (Supplementary Table 3). Taken together, these results indicated that TGMS lines with *TMS5* targeted editing using the CRISPR/Cas9 system could be applied to improve production in hybrid rice breeding.

## Discussion

In recent years, RNA interference (RNAi) and antisense approaches have become more commonly used in reverse genetics systems^40^. In our previous work, we knocked down *TMS5* expression using RNAi. Although homozygous TGMS plants were generated, sterile heterozygotes under high temperatures were simultaneously obtained^15^. Therefore, sterile heterozygous TGMS plants cannot be used for hybrid rice breeding. We then knocked down *TMS5* expression using an antisense RNA technique and obtained very low rates of TGMS plants with a relatively high CSIT (Supplementary Figure 8), potentially due to incomplete suppression of *TMS5*. This result suggested that both RNAi and antisense RNA technology provide sequence-dependent post-transcriptional inhibition of gene expression, resulting in incomplete depletion of target genes^40^. Nevertheless, TGMS lines with low CSIT guarantee seed production in two-line hybrid rice breeding^43^. Importantly, using a modified sgRNA to cleave a target gene exon via DSBs and nonhomologous end-joining (NHEJ) repair in the CRISPR/Cas9 system, knockout genotypes can be deliberately generated in plants. CRISPR/Cas9-mediated site-directed mutagenesis produced a loss of function of *TMS5* and generated TGMS rice with low and stable CSIT (Fig. 5). Moreover, regardless of the presence of the CRISPR/Cas9 transgene, the target gene mutations could be stably transmitted to future generations^41^. Therefore, “transgene clean” mutants could be obtained in the T_1_ generation (Figs 2, 3 and 5 and Supplementary Figure 2). In summary, the mutations generated using this strategy are essentially similar to the spontaneous or induced mutations that occur in conventional breeding, in addition to the advantage that the objective trait of CRISPR/Cas9 editing in plants can be deliberately improved^39^.

Currently, hybrid breeding is an important approach for the improvement of rice yield in China^6^. Several years or decades were required to obtain and select sterile lines under specific temperature and light conditions in the two-line breeding system^21^,^22^,^23^,^24^. In contrast, only one year was required to produce “transgene clean” *tms5* TGMS plants in the T_1_ generation using the CRISPR/Cas9-mediated site-directed mutagenesis system. Commercial sterile lines are mainly produced using the available sterile lines as the sterile gene donor (female parent) in traditional cross and backcross breeding programs^21^. However, there are limited genetic resources for the intensive use of a single source of male-sterile cytoplasm, and variety collapse of a source of sterile cytoplasm is considered to be disastrous in developing hybrids^44^. Therefore, by developing TGMS lines in different genetic backgrounds, the genetic diversity—especially the cytoplasm diversity—can be increased, improving the efficiency of the exploitation of heterosis. The methodology applied in our work to produce TGMS lines will mitigate limitations related to traditional hybrid breeding.

The production of hybrid rice using the three-line system involves CMS, maintainer and restorer lines. Commercial CMS lines can be mainly categorized into three groups: wild-abortive (WA), Honglian (HL) and Baotai (BT)^45^. The utilization of different types of three-line CMS lines is restricted because special CMS-restorer genes are required from the male parents, and the germplasms of maintainers and restorers are not abundant. For example, approximately 5% of the rice germplasms can serve as restorer lines in China, Southeast Asia and America^10^,^46^,^47^. The CMS and maintainer lines possess the same nucleus but different cytoplasms^8^. In the present study, we used CRISPR/Cas9-mediated editing of nuclear genes encoding *TMS5* in maintainer lines to obtain TGMS lines with extremely similar characteristics to their corresponding CMS lines. Almost all of the normal varieties with or without specific CMS-restorer genes could restore the fertility of TGMS lines^12^,^14^. This method retains all of the merits of CMS lines, avoids the problems caused by limited genetic resources and improves the heterosis utilization efficiency. Therefore, we termed this direct and efficient TGMS breeding method as “turning three-line into two-line”.

The breeding of *japonica* hybrid rice has not been widely applied due to a lack of male-sterile lines with high agronomic value. The *japonica* hybrid rice occupies approximately only 3% of the *japonica* rice planting area in China^10^,^48^. The promotion of *japonica* hybrid rice breeding will be a breakthrough in rice production in China. The sterility gene in *japonica* P/TGMS lines has been derived mainly from Nongken58S by employing a single genetic background, and it possesses the characteristics of high CSIT^43^. Nevertheless, it will be very convenient to use CRISPR/Cas9-mediated *TMS5* editing to cultivate sterile lines of *japonica* or wide-compatibility sterile line breeding to improve *japonica* or *japonica*-*indica* hybrid rice breeding^48^.

In two-line hybrid breeding, the cultivation of sterile lines with low CSIT is the key for ensuring the purity of hybrid seeds^43^. We tested CSITs of 7 TGMS lines generated using the TMS5ab editing system (Fig. 5) and found that varieties with different genetic backgrounds possessed different CSITs and that most of them met the requirements of breeding programs. These results suggested that the CSIT of *tms5* TGMS lines was determined according to their genetic backgrounds but not *tms5*. Furthermore, TGMS lines with higher CSITs could be crossed with lower CSIT lines to select new TGMS lines with lower CSITs.

In the present study, we selected 10 target sequences in the *TMS5* coding region to examine the efficiency of inducing target site editing and found that the efficiency of TMS5b was highest (up to 88.2%). Among the five constructs, the editing efficiency of TMS5ab was as high as 94.1%, and the homozygous rate of one of the target sequences was as high as 32.4%. These findings showed that the production of “transgene clean” TGMS plants was very convenient using the CRISPR/Cas9 system in the T_1_ generation and that the breeding efficiency could be markedly improved. Furthermore, the editing efficiency of the CRISPR/Cas9 system was affected by several factors. As previously reported^25^,^26^, targets with higher GC contents demonstrated relatively higher editing efficiencies (Fig. 1A and Supplementary Figure 3). In addition, as previously described^26^,^49^, a more stable secondary structure resulted in imperfect complementary base pairing of the target sequence and the target site (Fig. 1A and Supplementary Figure 4). As a result, the target sequence with a less stable secondary structure with sgRNAs tends to be selected in this system^49^. We also noted that the editing efficiency of the same target sequence was altered in different rice varieties (Fig. 1C). This observation may be due to the reduced activity of the CRISPR/Cas9 editing system in some varieties or to the higher percentage of faultless repairs after DSB^25^. The sgRNA expression level might be the limiting factor for target site editing in Arabidopsis^26^. However, when the sgRNA and *Cas9* expression levels reached certain thresholds, their expression may not be relevant to the target site editing efficiency in rice.

In the present study, we designed 10 target sequences in the *TMS5* gene and selected the most optimal TMS5ab construct for the development of commercial TGMS lines by comparing the editing efficiency and analysing the off-target efficiency of the target sequences. Using the TMS5ab construct, the *TMS5* gene was edited in 11 elite ice lines to develop the “transgene clean” TGMS lines within only one year. This method can shorten the breeding period, reduce the working complexity and cost, and facilitate the exploitation of heterosis. Our work will greatly promote the breeding process of TGMS lines and will maximize the number of cross combinations of three-line sterile lines, which will facilitate the development of hybrid rice breeding. This work can be applied for not only hybrid rice breeding but also potentially breeding of other hybrid crops.

## Methods

### Plant materials, growth conditions and generation of transgenic plants

TMS5as was derived from zhonghua11 (ZH11) by knocking down *TMS5* using anti-sense RNA technology. TMS5abS, TMS5cdS, TMS5efS, TMS5ghS and TMS5ijS were derived from ZH11 by the transformation of TMS5ab, TMS5cd, TMS5ef, TMS5gh, and TMS5ij vectors, respectively, via *Agrobacterium*-mediated transformation. ZS97BS, HNBS, TFBS, YXBS, ReBS, HHBS, ZZB, YJSMS, YNSMS, WSSMS, and GAZS were derived from the transformation of the TMS5ab vector using Zhenshan97B (ZS97B), HuanongB (HNB), TianfengB (TFB), YixiangB (YXB), ReB, HuahuiB (HHB), ZhongzheB (ZZB), Yuejingsimiao (YJSM), Yuenongsimiao (YNSM), Wushansimiao (WSSM), and GAZ as hosts, respectively. ZH11 and GAZ are conventional *Japonica* rice; Zhenshan97B (ZS97B), HuanongB (HNB), TianfengB (TFB), YixiangB (YXB), ReB, HuahuiB (HHB) and ZhongzheB (ZZB) are “three-line” *indica* maintainer lines. Yuejingsimiao (YJSM), Yuenongsimiao (YNSM), and Wushansimiao (WSSM) are conventional *indica* rice. Transgenic plants were generated using *Agrobacterium*-mediated transformation as previously described^13^,^15^. Unless indicated, all rice plants were grown in the field at the South China Agricultural University, Guangzhou (23_N, 113_E), during normal rice growing seasons, or in a growth chamber at DATs of 22, 24, 26, 28 and 30 °C with three replicates as previously described^15^.

### Vector constructs

The TMS5as vector was constructed as previously described^50^. An antisense fragment from *TMS5* was amplified and cloned into pYLox vector^15^. CRISPR/Cas9 was constructed as previously described^26^. Briefly, according to the design principles of the target sequences in the CRISPR/Cas9 system, 19 to 20 bases upstream of the PAM motif were selected as candidate target sequences, and target sequences in *TMS5* were determined for TMS5a, TMS5b, TMS5c, TMS5d, TMS5e, TMS5f, TMS5g, TMS5h, TMS5i and TMS5j (Supplemental Table 4) by Blast analysis of the rice genome (http://blast.ncbi.nlm.nih.gov/Blast.cgi) to ensure that no other genes were targeted. Equivalent amounts of forward and reverse primers (0.05–0.1 μM) for one target were mixed and incubated at 90 °C for 1 min, followed by a gradual cool-down to room temperature to form the target adaptor. Approximately 1 μg each of the pYLgRNA-OsU6a and pYLgRNA-OsU3 vectors was digested using 10 U *Bsa* I (NEB), respectively. The ligation reaction (10 μl) contained 1 μ1 of 10 x T4 DNA ligase buffer, 20 ng of pYLgRNA-OsU6a or pYLgRNA-OsU3 vector, 1 μ1 of the target sequence adaptor, and 35 U of T4 DNA ligase (Takara, Dalian, China). The target sequence adaptors TMS5a, TMS5c, TMS5e, TMS5g and TMS5i, respectively, were ligated into the linearized pYLgRNA-OsU6a vector (digested with 10 U of *Bsa* I), and TMS5b, TMS5d, TMS5f, TMS5h, and TMS5j, respectively, were cloned into pYLgRNA-OsU3 to generate sgRNA expression cassettes with target sequences. The sgRNA expression cassettes with the target sequences TMS5a, TMS5c, TMS5e, TMS5g and TMS5i were amplified from pYLgRNA-OsU6a using primers Pps-GGL and Pgs-GG2 and the sgRNA expression cassettes with target sequences TMS5b, TMS5d, TMS5f, TMS5h, and TMS5j were amplified from pYLgRNA-OsU3 using primers Pps-GG2 and Pgs-GGR with High-Fidelity DNA polymerase KOD-Plus (Toyobo, Osaka, Japan) over 30 cycles (94 °C, 30 s; 58 °C, 30 s; 68 °C, 20 s). The sgRNA expression cassettes with target sequences and binary plasmid pYLCRISPR/Cas9Pubi-H were digested with *Bsa* I and subsequently purified. Ten microliters of the reaction solution consisted of 1 μl of 10 x T4 DNA ligase buffer, 15 ng of purified sgRNA expression cassettes of TMS5a and TMS5b, 60 ng of pYLCRISPR/Cas9Pubi-H, and 70 U of T4 DNA ligase (Takara, Dalian, China). The TMS5ab vector was constructed by ligating sgRNA expression cassettes of TMS5a and TMS5b to the binary plasmid pYLCRISPR/Cas9Pubi-H. The same procedures were used for the TMS5cd, TMS5ef, TMS5gh, and TMS5ij constructs as for TMS5ab. The relevant PCR primers are listed in Supplementary Table 4.

### Mutation detection and analysis of transgenic plant lines

To determine the mutation of target sites, genomic DNA from the leaves of transgenic rice plants was extracted using the sodium dodecyl sulphate method^51^. PCR amplifications were performed using primer pairs (Supplemental Table 4) surrounding the designed target sites. The PCR products were directly sequenced or cloned into the pEASY-Blunt (TransGen Biotech, Beijing, China) vector and sequenced using the Sanger method. Mutations were identified by comparing the sequences of transgenic and WT plants. The sequencing chromatograms from mutations were analysed, and mutations containing normal sequencing chromatograms were considered homozygotes. Mutations containing superimposed sequencing chromatograms were considered heterozygous or biallelic mutations. Heterozygous sequences from direct sequencing were decoded using the degenerate sequence decoding method^52^. The relevant PCR primers are listed in Supplementary Table 4.

The identification of “transgene clean” plants was conducted using T_1_ progeny of T_0_ homozygous mutations. The transgenic lines were analysed by PCR using *HPT* and *Cas9* primers and agarose gel electrophoresis. The pYLCRISPR/Cas9Pubi-H plasmids and ZH11 DNA were selected as positive and negative controls, respectively. *HPT*- and *Cas9*-negative plants were considered “transgene clean” plants. Some “transgene clean” plants identified by PCR were further confirmed by Southern blotting. Southern blots were performed using a DIG-High Prime DNA Labeling and Detection Starter Kit I (Roche Diagnostics, Mannheim, Germany) according to a standard program supplied by the manufacturer. In brief, PCR products of *HPT* were labelled with GIG-dUTP by random priming (Roche Diagnostics) and used as hybridization probes. Genomic DNA from the leaves of rice plants was extracted using the Cetyltrimethylammonium bromide (CTAB) method. Genomic DNA was digested overnight using *Eco*RI (Takara, Dalian, China) and then separated by 0.8% agarose electrophoresis and transferred onto Hybond N^+^ nylon membranes (Amersham Bioscience, Bucks, UK) using the capillary transfer method. Hybridization was performed at 65 °C in DIG Easy Hyb buffer. The membranes were washed twice with wash buffer (2 x SSC, and 0.1% SDS) at 68 °C and then blocked with blocking solution and incubated with anti-digoxigenin-AP solution for 30 min. Subsequently, the membranes were washed twice with wash buffer (0.1 M maleic acid, 0.15 M NaCl, 0.3% (v/v) Tween 20, pH 7.5) at room temperature for 15 min each, followed by equilibration with detection buffer (100 mM Tris-Cl, 100 mM NaCl, pH 9.5) for 2–5 min. One millilitre of CSPD ready-to-use solution was added to the membranes to stimulate chemiluminescence. The chemiluminescence signals were detected and analysed using X-ray films.

### Expression analyses

Total RNA was isolated from the leaves or panicles of T_0_ rice plants using TRIzol (Invitrogen, Carlsbad, CA, USA). DNase I-treated total RNA (2.0 μg) was used for reverse transcription using an M-MLV-RT kit (Takara, Dalian, China) with a mixture of oligo(dT) and sgRNA RT primers (Supplemental Table 4). Real-time quantitative PCR was performed with gene- and target-specific primers (Supplemental Table 3) using the SsoFast™ EvaGreen Supermix kit (Bio-Rad, USA) and CFX96 Real-Time PCR Detection System (Bio-Rad) as previously described^15^. Three replicates were evaluated for each gene. The internal standard gene *OsActin1* was used to normalize the cDNA levels of the target genes. Total protein was extracted from rice panicles, and immunoblot analysis was performed using a previously described method^15^. HSP82^53^ (Code: AbM51099-31-PU; BPI, China) was used as the internal standard protein. The relevant PCR primers are listed in Supplementary Table 4.

### RNA secondary structure analysis

Secondary structure analysis of target-sgRNA sequences was performed with the RNA Folding Form (http://unafold.rna.albany.edu/?q=mfold/RNA-Folding-Form).

### Characterization of phenotypes

Rice florets were photographed using an OLYMPUS DP70 digital camera under an OLYMPUS SZX10 dissecting microscope. Mature pollen grains were stained using 1% I_2_–KI solution and photographed using an OLYMPUS BX51 or Leica DNRXA microscope.

## Additional Information

**How to cite this article**: Zhou, H. *et al*. Development of Commercial Thermo-sensitive Genic Male Sterile Rice Accelerates Hybrid Rice Breeding Using the CRISPR/Cas9-mediated *TMS5* Editing System. *Sci. Rep.* **6**, 37395; doi: 10.1038/srep37395 (2016).

**Publisher’s note:** Springer Nature remains neutral with regard to jurisdictional claims in published maps and institutional affiliations.

## Supplementary Material

Supplementary Information

## Figures and Tables

**Table 1:**
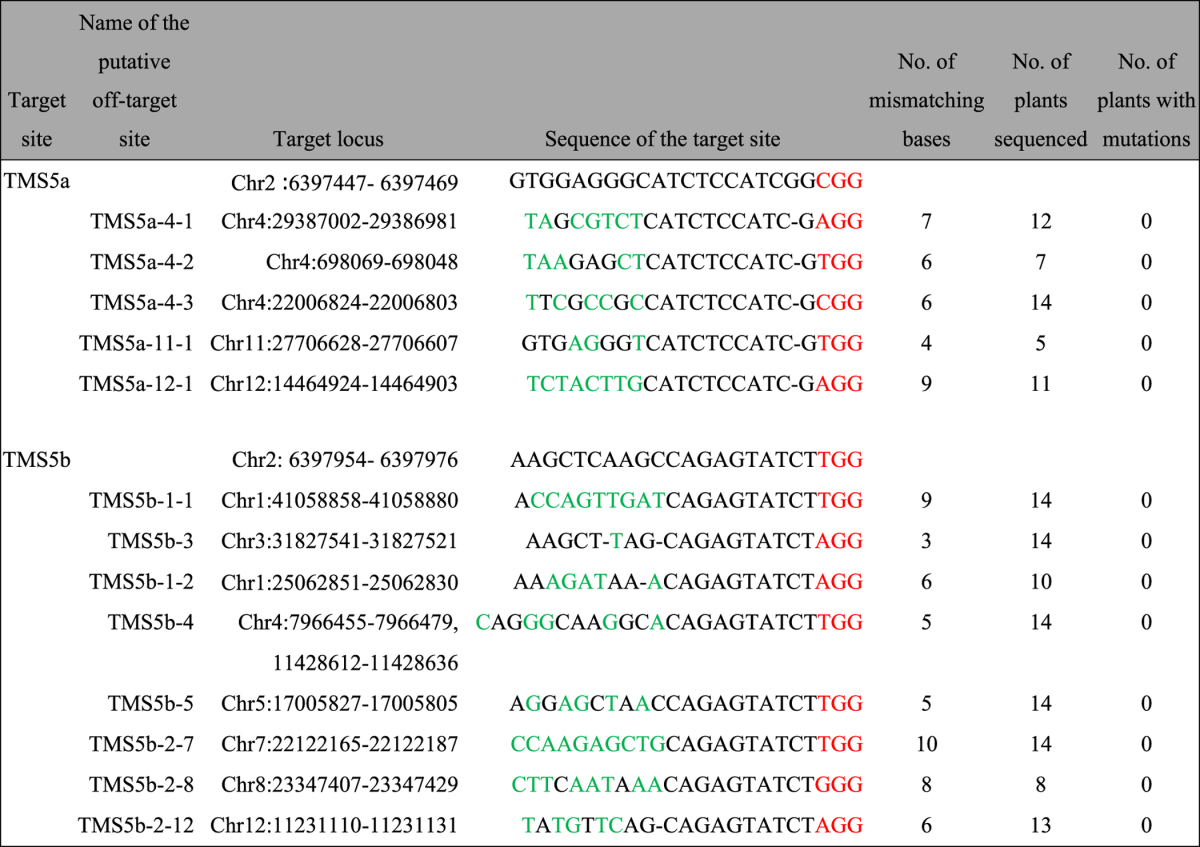
Mutations detected in the putative off-target sites of the TMS5a and TMS5b target sites.

Nucleotides in red represent the PAM motif of each target site. Nucleotide deletions are shown as –. Nucleotides that are substituted are shown in green.
