# Supplementary material for: Development of Commercial Thermo-sensitive Genic Male Sterile Rice Accelerates Hybrid Rice Breeding Using the CRISPR/Cas9-mediated TMS5 Editing System
Source: Sci Rep. 2016 Nov 22;6:37395. doi: 10.1038/srep37395 (PMC5118805; doi:10.1038/srep37395)
Supplement: Supplementary Information [file srep37395-s1.pdf]

SUPPLEMENTARY INFORMATION

Development of Commercial Thermo-sensitive Genic Male Sterile Rice Accelerates Hybrid Rice Breeding Using the CRISPR/Cas9-mediated *TMS5* Editing System

Hai Zhou[1, 2, 3], Ming He[1, 2, 3], Jing Li[1, 2, 3], Liang Chen[1, 2, 3], Zhifeng Huang[1, 2, 3], Shaoyan Zheng[1, 2, 3], Liya Zhu[1, 2, 3], Erdong Ni[1, 2, 3], Dagang Jiang[1, 2, 3], Bingran Zhao[4], Chuxiong Zhuang[1, 2, 3, †]

[1]State Key Laboratory for Conservation and Utilization of Subtropical Agro-bioresources, Guangzhou 510642, China

[2]Key Laboratory of Plant Functional Genomics and Biotechnology of Guangdong Provincial Higher Education Institutions, Guangzhou 510642, China

[3]College of Life Sciences, South China Agricultural University, Guangzhou 510642, China

[4]State Key Laboratory of Hybrid Rice, China National Hybrid Rice R&D Center, Changsha 410125, China

†Correspondence should be addressed to C.Z. (zhuangcx@scau.edu.cn)

SUPPLEMENTARY FIGURES 1-8

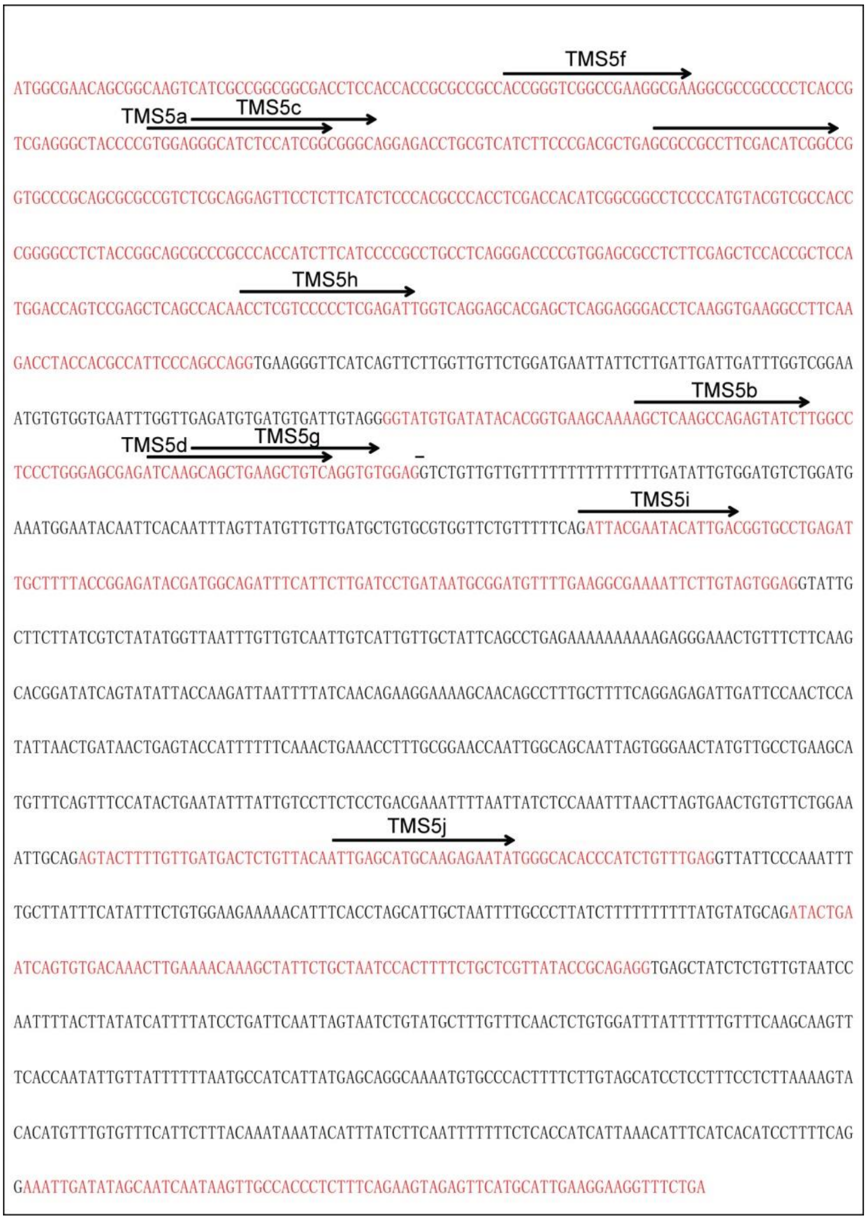

Supplementary Figure 1. Ten target sites in *TMS5*.

The exons and introns of *TMS5* are shown in red and black, respectively.

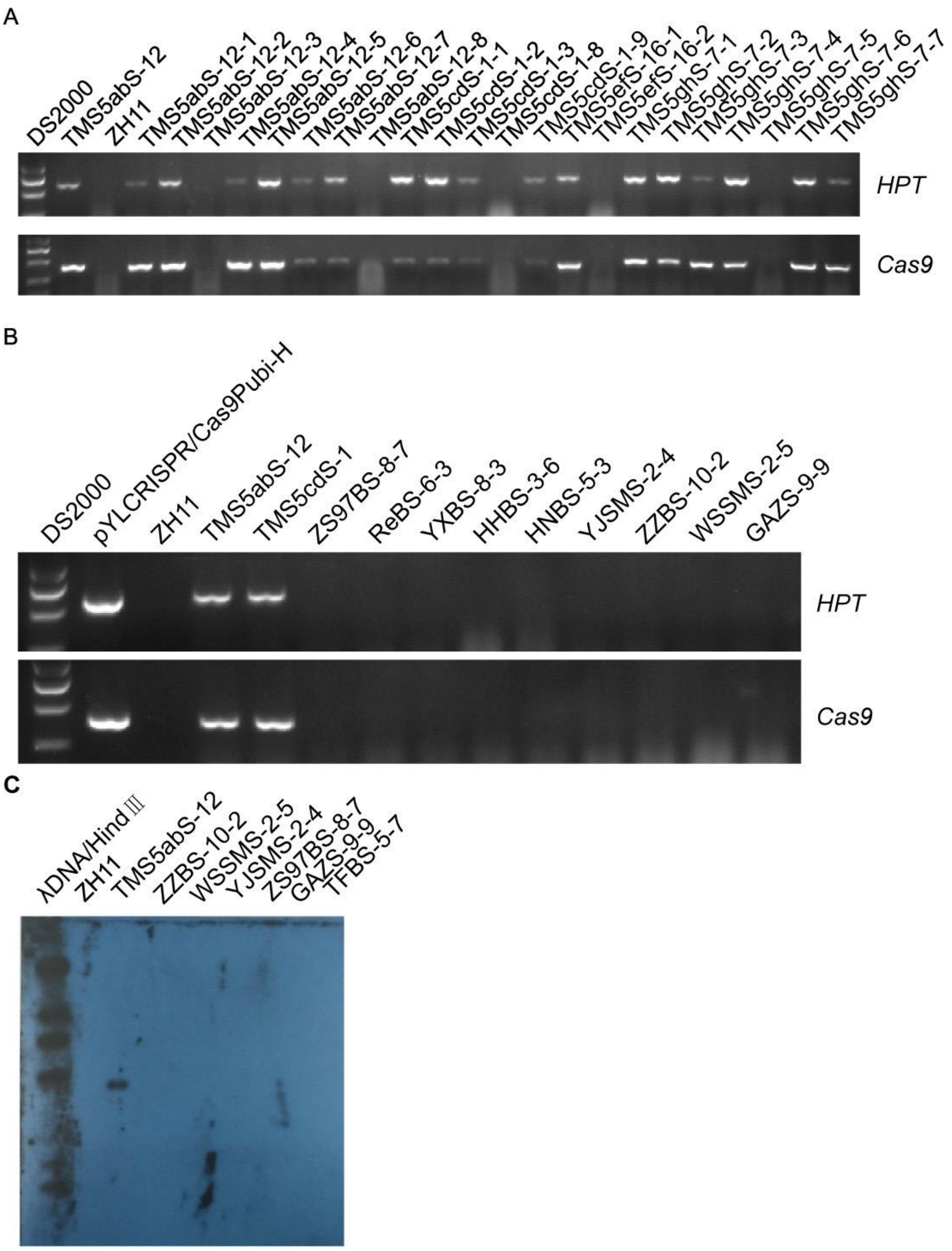

Supplementary Figure 2. Detection of *hygromycin phosphotransferase* (*HPT*) and *Cas9* in T_1 generation plants.

(A and B) *HPT* and Cas9 are the selection markers for transgenic rice. (A) Detection of *HPT* and *Cas9* in T_1 generation plants of TMS5abS-12, TMS5cdS-1, TMS5efS-16 and TMS5ghS-7 using PCR. The wild type, ZH11, was a negative control, and TMS5abS-12 was a positive control for the transgene. TMS5abS12-1, 2, 3, 4, 5, 6, 7 and 8 are T_1 generation plants of TMS5abS-12. TMS5cdS1-1, 2, 3, 8 and 9 are T_1 generation plants of TMS5cdS-1. TMS5efS-16-1 and 2 are T_1 generation plants of

TMS5efS-16. TMS5ghS-7-1, 2, 3, 4, 5, 6 and 7 are T_1 generation plants of TMS5ghS-7. (B) Detection of *HPT* and *Cas9* in ZS97BS-8-7, ReBS-6-3, YXBS-8-3, HHBS-3-6, HNBS-5-3, YJSMS-2-4, ZZBS-10-2, WSSMS-2-5, GAZS-9-9 and TFBS-5-7 using PCR. ZS97BS-8-7, ReBS-6-3, YXBS-8-3, HHBS-3-6, HNBS-5-3, YJSMS-2-4, ZZBS-10-2, WSSMS-2-5, GAZS-9-9 and TFBS-5-7 are the T_1 generation TGMS plants induced by the TMS5ab system in the ZS97B, ReB, YXB, HHB, HNB, YJSM, ZZB, WSSM, GAZ, and TFB backgrounds, respectively. (C) Detection of *HPT* in the T_3 generation plants ZZBS-10-2, WSSMS-2-5, YJSMS-2-4, ZS97BS-8-7, GAZS-9-9 and TFBS-5-7 by Southern blotting. TMS5abS-12 was a positive control for the transgene.

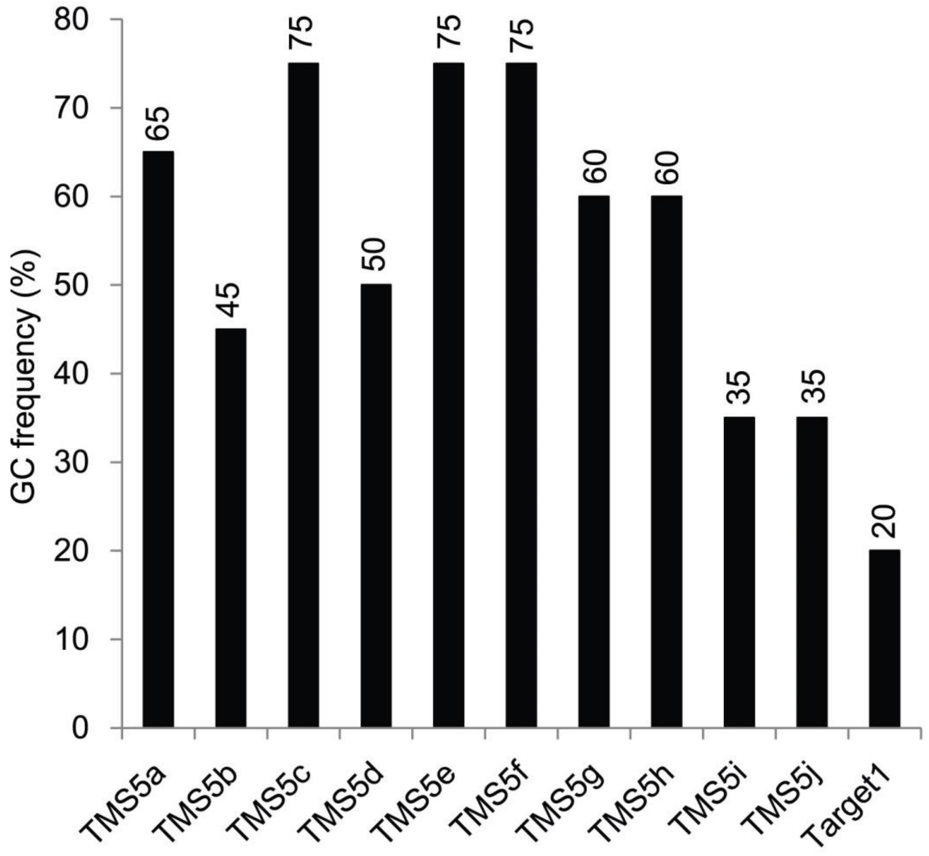

Supplementary Figure 3. GC contents of the 11 target sequences.

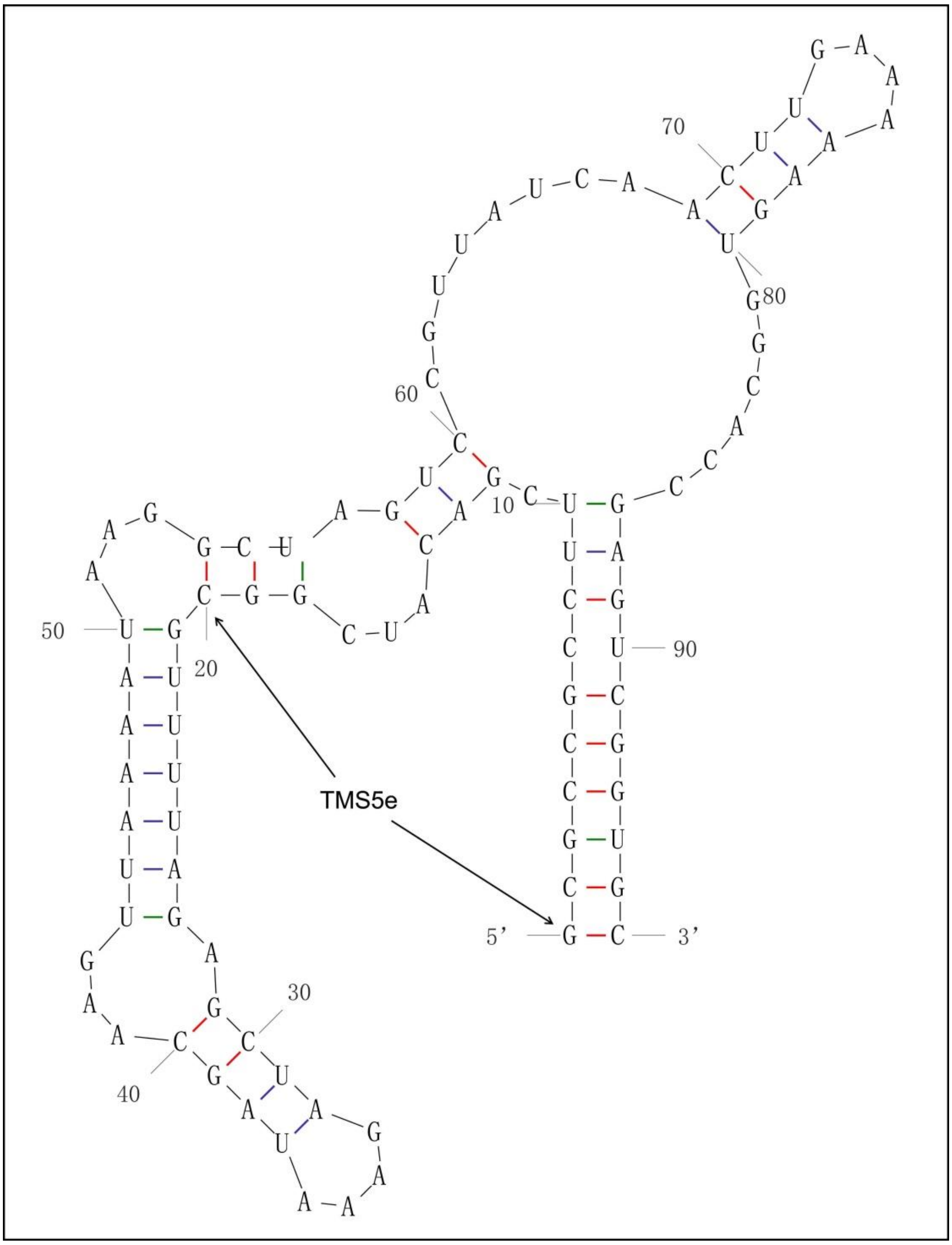

Supplementary Figure 4. TMS5e-sgRNA secondary structure.

The secondary structure of TMS5e-sgRNA was analysed using the RNA Folding Form (http://unafold.rna.albany.edu/?q=mfold/RNA-Folding-Form). The sequence between the two arrowheads indicates the TMS5e target sequence.

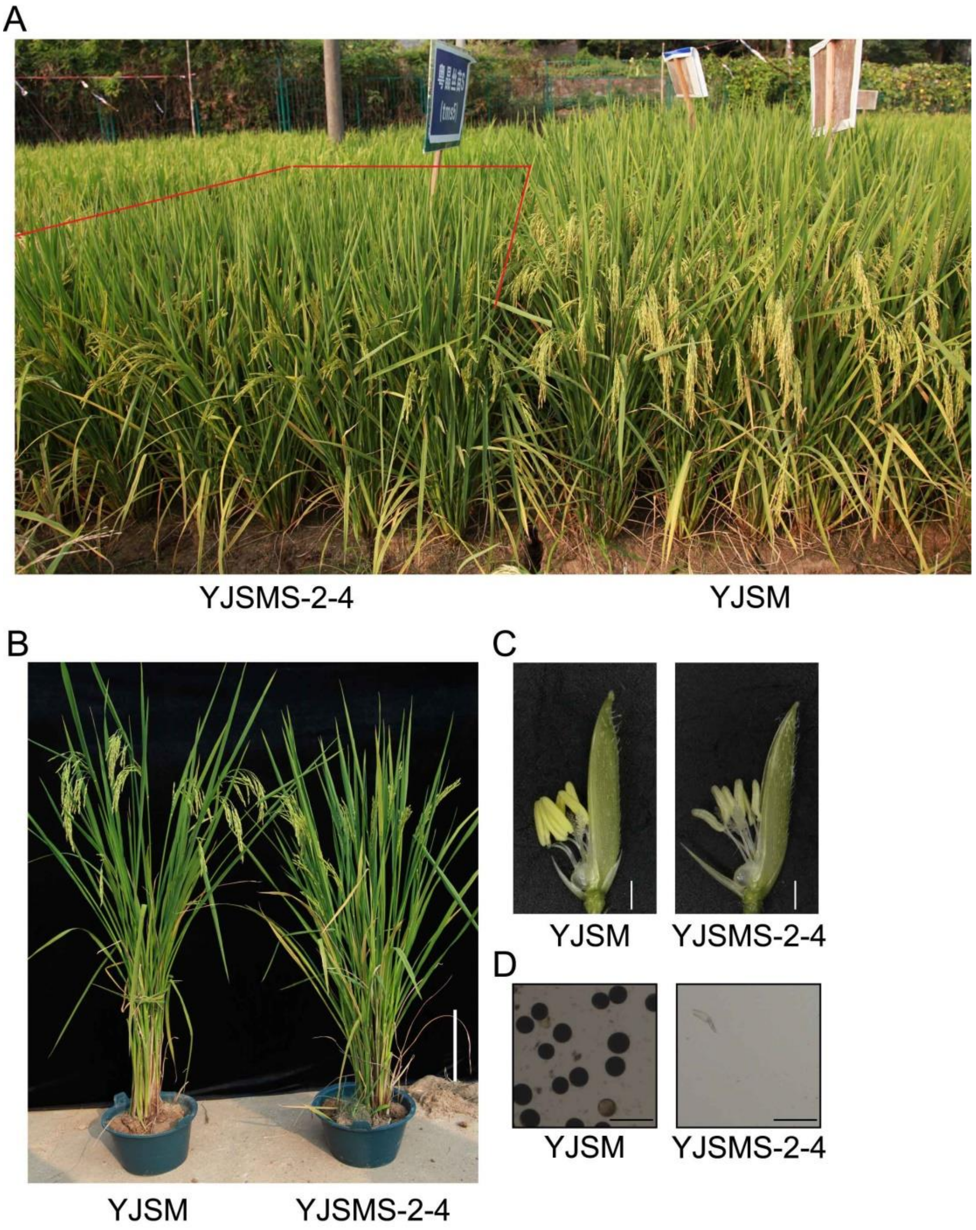

Supplementary Figure 5. Phenotypes of YJSMS at high temperature.

(A) Field performance of YJSMS and Yuejingshimiao (YJSM) populations in the experimental plots of the China National Hybrid Rice R&D Center (Changsha) in the summer of 2015. The YJSMS population was grown in the area within the red line. (B) Plant morphologies of YJSM and YJSMS at HT. (C) Anther phenotypes of YJSM and YJSMS at HT. (D) Pollen fertility levels of YJSM and YJSMS at HT. HT, high temperature; LT, low temperature. Scale bars, 20 cm in (B), 1 mm in (C), and 100 μm in (D).

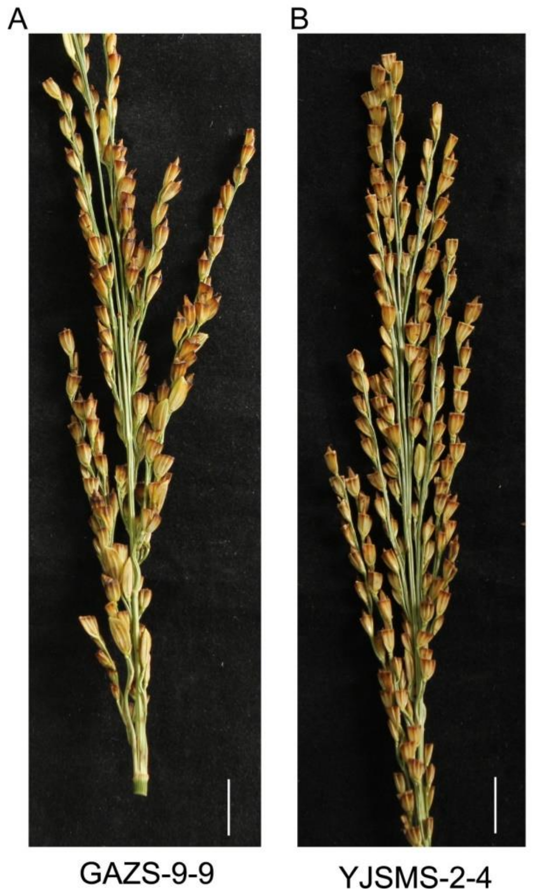

Supplementary Figure 6. Seed settings of GAZS and YJSMS after artificial pollination.

(A) Seed setting of GAZS-9-9 after pollination. (B) Seed setting of YJSMS-2-4 after pollination.

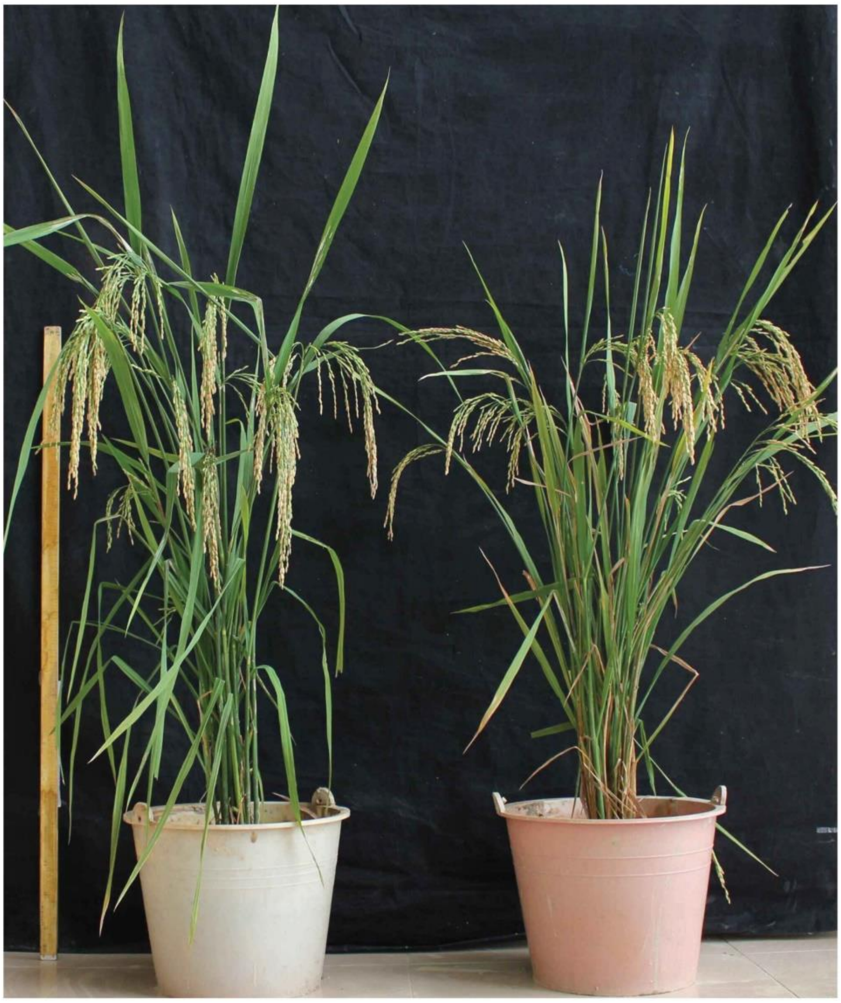

Supplementary Figure 7. Phenotypes of HNB and the hybrid of HNBS × HH1179.

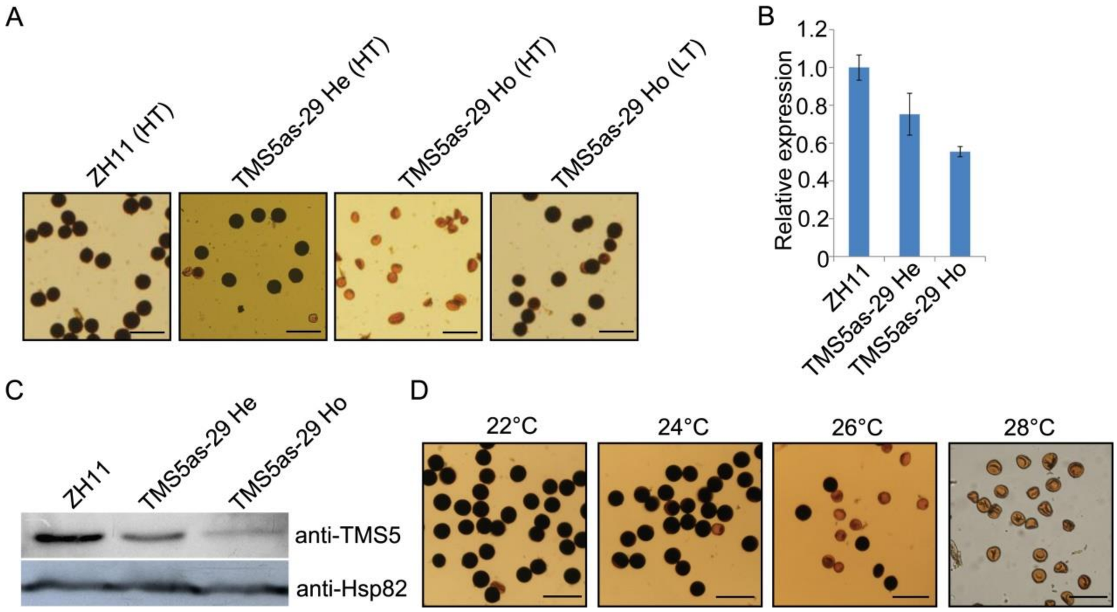

Supplementary Figure 8. Pollen fertility and the *TMS5* expression level in *TMS5* knockdown plants using the antisense RNA technique.

(A) Pollen fertility levels of ZH11 and *TMS5* antisense RNA plants (TMS5as-29He and Ho) at HT and LT. TMS5as-29He showed partial abortion, whereas TMS5as-29Ho displayed complete sterility at the HT. The fertility of TMS5as-29Ho was fully restored at the LT. He, heterozygote; Ho, homozygote. (B) Expression levels of *TMS5* in ZH11, TMS5as-29He and TMS5as-29Ho plants assessed by real-time PCR analysis. *TMS5* expression was slightly decreased in TMS5as-29He and TMS5as-29Ho plants compared with the ZH11. *OsActin1* was used as the internal control. (C) TMS5 relative expression levels in ZH11, TMS5as-29He and TMS5as-29Ho plants using immunoblot analysis. HSP82 was used as the reference protein. *TMS5* protein expression was lower in TMS5as-29Ho plants compared with TMS5as-29He plants. (D) Pollen fertility levels of TMS5as-29Ho plants grown at 22 ℃, 24 ℃, 26 ℃, and 28 ℃ under photoperiod conditions of 12 h of light and 12 h of darkness. Scale bars, 100 μm.

SUPPLEMENTARY TABLES 1-4

Supplementary Table 1. Target site sequences.

Target	Sequence of target sites
TMS5a	GTGGAGGGCATCTCCATCGGCGG
TMS5b	AAGCTCAAGCCAGAGTATCTTGG
TMS5c	GGGCATCTCCATCGGCGGGCAGG
TMS5d	ATCAAGCAGCTGAAGCTGTCAGG
TMS5e	GCGCCGCCTTCGACATCGGCCGG
TMS5f	ACCGGGTCGGCCGAAGGCGAAGG
TMS5g	GCAGCTGAAGCTGTCAGGTGTGG
TMS5h	AACCTCGTCCCCCTCGAGATTGG
TMS5i	GGAGATTACGAATACATTGACGG
TMS5j	ATTGAGCATGCAAGAGAATATGG
Target1	GATAAAAATTTTACTCTTGATGG

Nucleotides in red represent the PAM motif of each target site.

Supplementary Table 2. Segregation ratios of CRISPR/Cas9-mediated *TMS5* editing plants in the T_1 generation.

T_0 lines	T_1 segregation ratio
**GAZS-1**	**9:4**
**GAZS-9**	**24:5**
**YJSMS-2**	**14:6**
**YJSMS-31**	**16:9**
**ZS97BS-8**	**20:6**
**ZS97BS-108**	**18:3**
**YXBS-8**	**11:9**
**HHBS-3**	**14:6**
**HHBS-9**	**17:4**
**WSSMS-2**	**29:5**
**TFBS-5**	**16:8**
**ZZBS-10**	**16:4**
**ReBS-6**	**27:2**

Segregation ratio = *HPT*- and *Cas9*-positive plants : *HPT*- and *Cas9*-negative plants.

Supplementary Table 3. Yield components of HNB and the HNBS × HH1179 hybrid.

Rice	Length of main panicle (cm)	Increasing percentage (%)	Number of grains in main panicle	Increasing percentage (%)	Number of panicles	Increasing percentage (%)	Thousand seed weight (g)	Increasing percentage (%)
HNB	22.6±1.1	-	213±30	-	8.2±1.1	-	19.7±0.4	-
HNBS x HH1179	29.7±1.3	31.4**	315±18	47.9**	7.6±0.5	-7.3	23.6±0.3	19.8**

Rice	Number of plants per plot	Setting rate (%)	Increasing percentage (%)	Yield per plant (g)	Increasing percentage (%)	Yield per plot (g)	Increasing percentage (%)
HNB	36	88.4±2.9	-	20.9±0.5	-	753.3±10.4	-
HNBS × HH1179	36	82.2±0.9	-7**	23.9±0.1	14.4**	872.7±28.7	15.9*

T-tests were performed between HNB and the HNBS and HH1179 cross (* and ** indicate $P < 0.05$ and $P < 0.01$, respectively). There were three replicates for the plot and 15 replicates for the setting rate. All other assessments consisted of five replicates.

Supplementary Table 4. Primers used for DNA constructs, sequencing, and transcript analysis.

Primer name	Primer sequence (5'-3')	Purpose
U-F	CTCCGTTTTACCTGTGGAATCG	DNA constructs
gRNA-R	CGGAGGAAAATTCCATCCAC	DNA constructs
Pps-GGL	TTCAGAGGTCTCTCTCGCACTGGAATCGGCAGCAAAGG	DNA constructs
Pgs-GG2	AGCGTGGGTCTCGTCAGGGTCCATCCACTCCAAGCTC	DNA constructs
Pps-GG2	TTCAGAGGTCTCTCTGACACTGGAATCGGCAGCAAAGG	DNA constructs
Pgs-GGR	AGCGTGGGTCTCGACCGGGTCCATCCACTCCAAGCTC	DNA constructs
TMS5a F	GCCGTGGAGGGCATCTCCATCGG	DNA constructs
TMS5a R	AAACCCGATGGAGATGCCCTCCA	DNA constructs
TMS5b F	GGCAAGCTCAAGCCAGAGTATCT	DNA constructs
TMS5b R	AAACAGATACTCTGGCTTGAGCT	DNA constructs
TMS5c F	GCCGGGCATCTCCATCGGCGGGC	DNA constructs
TMS5c R	AAACGCCCGCCGATGGAGATGCC	DNA constructs
TMS5d F	GGCATCAAGCAGCTGAAGCTGTC	DNA constructs
TMS5d R	AAACGACAGCTTCAGCTGCTTGA	DNA constructs
TMS5e F	GCCGCGCCGCCTTCGACATCGGC	DNA constructs
TMS5e R	AAACGCCGATGTCGAAGGCGGCG	DNA constructs
TMS5f F	GGCACCGGGTCGGCCGAAGGCGA	DNA constructs
TMS5f R	AAACTCGCCTTCGGCCGACCCGG	DNA constructs
TMS5g F	GCCGCAGCTGAAGCTGTCAGGTG	DNA constructs
TMS5g R	AAACCACCTGACAGCTTCAGCTG	DNA constructs
TMS5h F	GGCAACCTCGTCCCCCTCGAGAT	DNA constructs
TMS5h R	AAACATCTCGAGGGGGACGAGGT	DNA constructs
TMS5i F	GCCGGAGATTACGAATACATTGA	DNA constructs
TMS5i R	AAACTCAATGTATTCGTAATCTC	DNA constructs
TMS5j F	GGCATTGAGCATGCAAGAGAATA	DNA constructs
TMS5j R	AAACTATTCTCTTGCATGCTCAA	DNA constructs
Target1 F	GCCGATAAAAATTTTACTCTTGA	DNA constructs
Target1 R	AAACTCAAGAGTAAAATTTTTAT	DNA constructs
TMS5i F	AAAAGGATCCGTCACACTGATTCAGTATCTC	DNA constructs
TMS5i R	AAAAAAGCTTCATGGACCAGTCCGAGCTC	DNA constructs
RNAi-Mlu	CACCCTGACGCGTGGTGTTACTTCTGAAGAGG	DNA constructs
RNAi-Pst	ACTAGAACTGCAGCCTCAGATCTACCATGGTCG	DNA constructs
TMS5as F	AAAAAAAGCTTTGACAGCTTCAGCTGCTTG	DNA constructs
TMS5as R	AAAAAACTAGTATGGCGAACAGCGGCAAG	DNA constructs
TMS5-4 F	CGTGTTCACCCTTCCAACCT	DNA sequencing
TMS5-4 R	GCTCGTGCTCCTGACCAATC	DNA sequencing
TMS5-5 F	CAGGAGTTCCTCTTCATCTC	DNA sequencing
TMS5-5 R	AGGCACCGTCAATGTATTCG	DNA sequencing
TMS5-6 F	GTTGATGCTGTGCGTGGTTC	DNA sequencing
TMS5-6 R	GGAATAACCTCAAACAGATGG	DNA sequencing

TMS5a-4-1 F	CACGAGATGCATGTGAGAAC	DNA sequencing
TMS5a-4-1 R	CAGGCCTATGATTCTAGGAG	DNA sequencing
TMS5a-4-2 F	GACTTCTAAGATACGGAAGAG	DNA sequencing
TMS5a-4-2 R	TGAACCAGGTTCGACATAGG	DNA sequencing
TMS5a-4-3 F	CCAGTCAACTACTCAAGTCC	DNA sequencing
TMS5a-4-3 R	TGGAGATGGAGCTGAGCTGA	DNA sequencing
TMS5a-11-1 F	CAACATCATCAATCCGAATCC	DNA sequencing
TMS5a-11-1 R	TGAGGTACCGATACCTCGTG	DNA sequencing
TMS5a-12-1 F	GCTACGTTCTCATCGTCTCG	DNA sequencing
TMS5a-12-1 R	CAGGAATCAGTAATGCACTGG	DNA sequencing
TMS5b-1-1 F	CGATCTTGATGATCTCACTCG	DNA sequencing
TMS5b-1-1 R	CACTCGTTAGTCAACTGGAAC	DNA sequencing
TMS5b-3 F	GAAATGATGGGTACAGTGTGG	DNA sequencing
TMS5b-3 R	GAGGAGAACGATGACGTTGG	DNA sequencing
TMS5b-1-2 F	TCGGTGTTGTGGTGAATCAG	DNA sequencing
TMS5b-1-2 R	TCGTCGTGCTATCCATGCAC	DNA sequencing
TMS5b-4 F	ACATGGTAGTGCTCACATGC	DNA sequencing
TMS5b-4 R	GTGTTGTCCTTGGTCTTGAC	DNA sequencing
TMS5b-5 F	GTCTGATCCATTCCGTCATG	DNA sequencing
TMS5b-5 R	CAACACTGTCACTAACACGTC	DNA sequencing
TMS5b-7 F	GAGCAAGTCTCATCTCACTG	DNA sequencing
TMS5b-7 R	TCTCTTGAGATGCTGTGGTC	DNA sequencing
TMS5b-8 F	GTTACTCCGTGGTACTAAGC	DNA sequencing
TMS5b-8 R	GAGTCATACATCCACGACTC	DNA sequencing
TMS5b-12 F	CAAGGCACAGATTGATGGAG	DNA sequencing
TMS5b-12 R	GGTAGTAGGGTAAATGAGACG	DNA sequencing
TMS5q F	TCTGCTAATCCACTTTTCTGCTC	RT-qPCR
TMS5q R	CCTTCCTTCAATGCATGAACTCT	RT-qPCR
OsActin1q F	CACATTCCAGCAGATGTGGA	RT-qPCR
OsActin1q R	GCGATAACAGCTCCTCTTGG	RT-qPCR
sgRNAq F	GTTTTAGAGCTAGAAATAGCAAG	RT-qPCR
sgRNAq R	CGACTCGGTGCCACTTTTTC	RT-qPCR
sgRNA RT	AAAAAGCACCGA	RT-qPCR
CAS9q F	ATCGACAGGAAGCGTTACAC	RT-qPCR
CAS9q R	CTGGGAAAGGTCGATACGAG	RT-qPCR
HPT F	GTCCGTCAGGACATTGTTGGAG	Genotyping
HPT R	GTCTCCGACCTGATGCAGCTCCGG	Genotyping
Cas9 F	CTCTTCCTTCCAAGTACGTG	Genotyping
Cas9 R	GAAAGGTCGATACGAGTCTC	Genotyping
